# Risk Factors and Prognosis in Patients With Anti-N-Methyl-D-Aspartate Receptor Encephalitis Requiring Prolonged Mechanical Ventilation

**DOI:** 10.3389/fneur.2022.814673

**Published:** 2022-02-09

**Authors:** Jingfang Lin, Qu Xiang, Xu Liu, Jinmei Li

**Affiliations:** ^1^Department of Neurology, West China Hospital, Sichuan University, Chengdu, China; ^2^West China Biomedical Big Data Center, West China Hospital, Chengdu, China

**Keywords:** anti-N-methyl-D-aspartate receptor encephalitis, mechanical ventilation, non-invasive ventilation, prolonged mechanical ventilation, functional outcomes

## Abstract

**Background:**

Mechanical ventilation (MV) is commonly used in anti-N-methyl-D-aspartate receptor (NMDAR) encephalitis patients with serious conditions. However, little is known about the potential risk factors and long-term outcomes of anti-NMDAR encephalitis requiring MV, especially prolonged MV.

**Methods:**

The data collected prospectively from 305 patients with anti-NMDAR encephalitis were retrospectively reviewed. The functional outcome was assessed using a modified Rankin scale (mRS) every 3 months.

**Results:**

We identified 62 (20.3%) patients who required MV. The most common reasons for MV were decreased consciousness and/or status epilepticus (SE). Among 60 patients analyzed, 27 patients required prolonged MV (>15 days). Prolonged MV primarily was based on the younger age, coma, tumor, and severe pneumonia. During the follow-up (median: 28 months, range: 3–87 months), 77% of patients required MV that exhibited a good outcome. In univariate analysis, prolonged MV, higher levels of C-reactive protein (CRP), and neutrophil-to-lymphocyte ratio (NLR) were found to be associated with poor neurological outcome at 6 months. Although the prolonged MV group exhibited a longer time to achieve a good outcome as compared to the short MV group (median duration 6 months vs. 3 months, *p* = 0.004), no significant difference was observed between the two groups about long-term outcomes.

**Conclusion:**

It is important to recognize that most anti-NMDAR encephalitis patients who required MV will achieve a favorable long-term outcomes, despite the longer duration of MV. Our results may help clinicians in the ventilator management of severe anti-NMDAR encephalitis patients.

## Introduction

Anti-N-methyl-D-aspartate receptor (NMDAR) encephalitis is a rare but severe autoimmune encephalitis (AE) (1, 2). During the acute phase of the disease, ~20% of the patients required mechanical ventilation (MV) (3). Previous studies have reported that MV (especially prolonged MV) can serve potentially as an independent risk factor for poor short-term outcomes in patients with AE (4). However, studies have yet to focus on the analysis of the clinical characteristics and long-term outcomes of anti-NMDAR encephalitis patients who required MV. In addition, although several cases have previously reported that severe form of anti-NMDAR encephalitis may require MV for several months or even years (5–7), the long-term outcomes in the subgroup of anti-NMDAR encephalitis patients who required prolonged MV have not been investigated before. It needs to be highlighted here that there is only limited data available on MV management in patients with anti-NMDAR encephalitis beyond available aggressive immunosuppression and oncological treatment options. The importance of prolonging MV treatment for patients with anti-NMDAR encephalitis should be emphasized. Because by prolonging MV treatment, patients can live and have the opportunity to receive a long enough course of specific treatment, which may be the reason for the good follow-up effect.

Thus, we specifically aimed to analyze the clinical characteristics of anti-NMDAR encephalitis patients who require MV. In addition, we further compared the clinical characteristics and outcomes between the prolonged MV patients and short-ventilated patients. We hypothesized that prolonged MV could be potentially associated with the long-term poor prognosis in anti-NMDAR encephalitis.

## Materials and Methods

### Patients

The data collected prospectively from the consecutive patients who visited the Department of Neurology at West China Hospital between June 2012 and September 2020 were retrospectively reviewed (the outcome of anti-NMDAR Encephalitis Study in Western China; registration number: ChiCTR1800019762) (8). All patients fulfilled the diagnosis of definite anti-NMDAR encephalitis according to the 2016 criteria (9). The inclusion criteria taken into the consideration were as follows: (1) rapid onset (<3 months) of one or more of the six following major groups of the symptoms: abnormal (psychiatric) behavior or cognitive dysfunction, speech disorders, seizures, movement disorder, decreased consciousness, autonomic instability, or central hypoventilation; (2) cerebrospinal fluid (CSF) showed the presence of anti-NMDAR antibodies by indirect immunofluorescence assays (IFAs; Euroimmun, Luebeck, Germany) (8); and (3) patients who required MV. The exclusion criteria used were as follows: (1) patients with alternative diagnoses (such infectious encephalitis, toxic-metabolic encephalopathy, or cerebral malaria, etc.); (2) patients who required MV due to the reasons other than anti-NMDAR encephalitis, such as after surgery; (3) patients with other positive neuronal antibodies, such as antibodies generated against contactin-associated protein-like 2, leucine-rich glioma-inactivated protein 1, gamma-aminobutyric acid receptors B1/B2 receptor, a-amino-3-hydroxy-5-methyl-4-isoxazol-propionic acid receptors 1 and 2, Dipeptidyl-peptidase–like protein 6, Hu, GAD65, Tr, Ma, PCA-2, Ri, CV2, Amphiphysin, and Yo; and (4) and patients with the missing data.

### Data Collection and Definition

The baseline data drawn from patients' standardized charts included (1) demographics, such as age and sex; (2) clinical features, such as reasons for MV, central hypoventilation, abnormal psychiatric or cognitive dysfunction, seizures, disturbance of consciousness, speech disorders, autonomic dysfunctions, and movement disorders; (3) laboratory and radiographic tests findings, such as CSF protein, pleocytosis (CSF cell count > 5 cells/mm^3^), abnormal electroencephalogram (EEG), abnormal MRI, C-reactive protein (CRP), the neutrophil-to-lymphocyte ratio in peripheral blood (NLR), interleukin-6 (IL-6), and procalcitonin (PCT) levels; and (4) other clinical features, such as the types of respiratory failure, the time to initiation of MV, the duration of MV, the presence of a tumor, admission intensive care unit (ICU), the time to initiation of immunotherapy treatment, and the length of stay in hospital.

The decision to use MV was assessed by the clinician in charge of the patient. However, MV was used routinely in the cases who fulfilled at least one of the following criteria (10–12): (1) decreased consciousness and/or status epilepticus (SE) that needs sedation; (2) severe hemodynamic instability; (3) cardiac arrest/arrhythmias; (4) persisting or worsening respiratory failure defined by at least two of the following signs: PaO_2_ <60 mmHg, PaCO_2_ >50 mmHg, inability to clear bronchial secretions or the respiratory rate ≥ 30 beats/min; (5) failure to achieve proper central hypoventilation; and 6) repeated coughing and aspiration after the swallowing.

The prolonged MV was defined as the period that required MV for more than 15 days based on the description in the previous studies (13, 14). In order to exclude bias, patients who died within 15 days after MV and for those the exact date of intubation or the duration of MV was not known were excluded from the analysis carried out to compare the characteristics between the prolonged MV patients and the short-ventilated patients. The respiratory failure was defined as arterial PO_2_ (PaO_2_) of <60 mmHg. However, if the corresponding arterial PCO_2_ (PaCO_2_) was <45 mmHg, the patient was classified as having Type I respiratory failure. A corresponding PaCO_2_ of more than 45 mmHg was defined as Type II respiratory failure (15). Ventilator-associated pneumonia (VAP) was defined as the presence of pneumonia that occurs ≥48 h after endotracheal intubation or ≤ 48 h after extubation (16).

Coma was defined based on the Glasgow Coma Scale (GCS) score ≤ 8 (17). SE was defined as continuous seizure activity >5 min or recurrent seizures without the complete recovery of the consciousness between the seizures that lasted longer than 5 min (18). Refractory status epilepticus (RSE) was defined as SE that did not respond to first- and second-line antiepileptic drugs (19). The first-line immunotherapy used included methylprednisolone (MP, 1,000 mg/day or 30 mg/kg/day for each course for 5 days), intravenous immunoglobulins (IVIg, 0.4 g/kg/day for each course for 5 days), or the plasma exchange (PE) treatment alone or as combination therapy (2). The second-line immunotherapy was defined as treatment given by administering rituximab, azathioprine, or cyclophosphamide (2, 20).

The functional outcome was ranked according to the modified Rankin scale (mRS) score ranging from 0 to 6, as follows: 0, healthy state; (1), minor symptoms, but able to carry out normal functions; (2), slight disability, but able to look after one's own affairs without assistance; (3), moderate disability requiring help for the various activities related to daily living but were able to walk; (4), severe disability requiring assistance while walking; (5), severe disability requiring constant nursing care; and (6), no longer alive. The recurrence was defined as the new onset or worsening of the symptoms occurring after at least 2 months of the stabilization or improvement (21).

### Follow-Up and Outcome

The follow-up was conducted every 3 months after the disease onset as assessed by the professional neurologist. The primary outcome was a good neurological outcome (mRS ≤ 2) (20). The secondary outcomes included mortality and recurrence.

### Statistical Analysis

The different categorical variables were calculated by Fisher's exact test or Chi-squared test appropriately. The continuous variables were determined by the Mann-Whitney *U* test or *t*-test based on their distribution. A two-side *p* < 0.05 was considered to be statistically significant. Kaplan-Meier survival analysis was used to analyze the time until patients reached their endpoint events. SPSS version 25.0, GraphPad Prism version 7, and R software version 3.6.1 were used for the statistical analyses. We did not correct for multiple comparisons because this was a study intended to generate hypotheses.

## Results

### Baseline Characteristics

**Figure 1:**
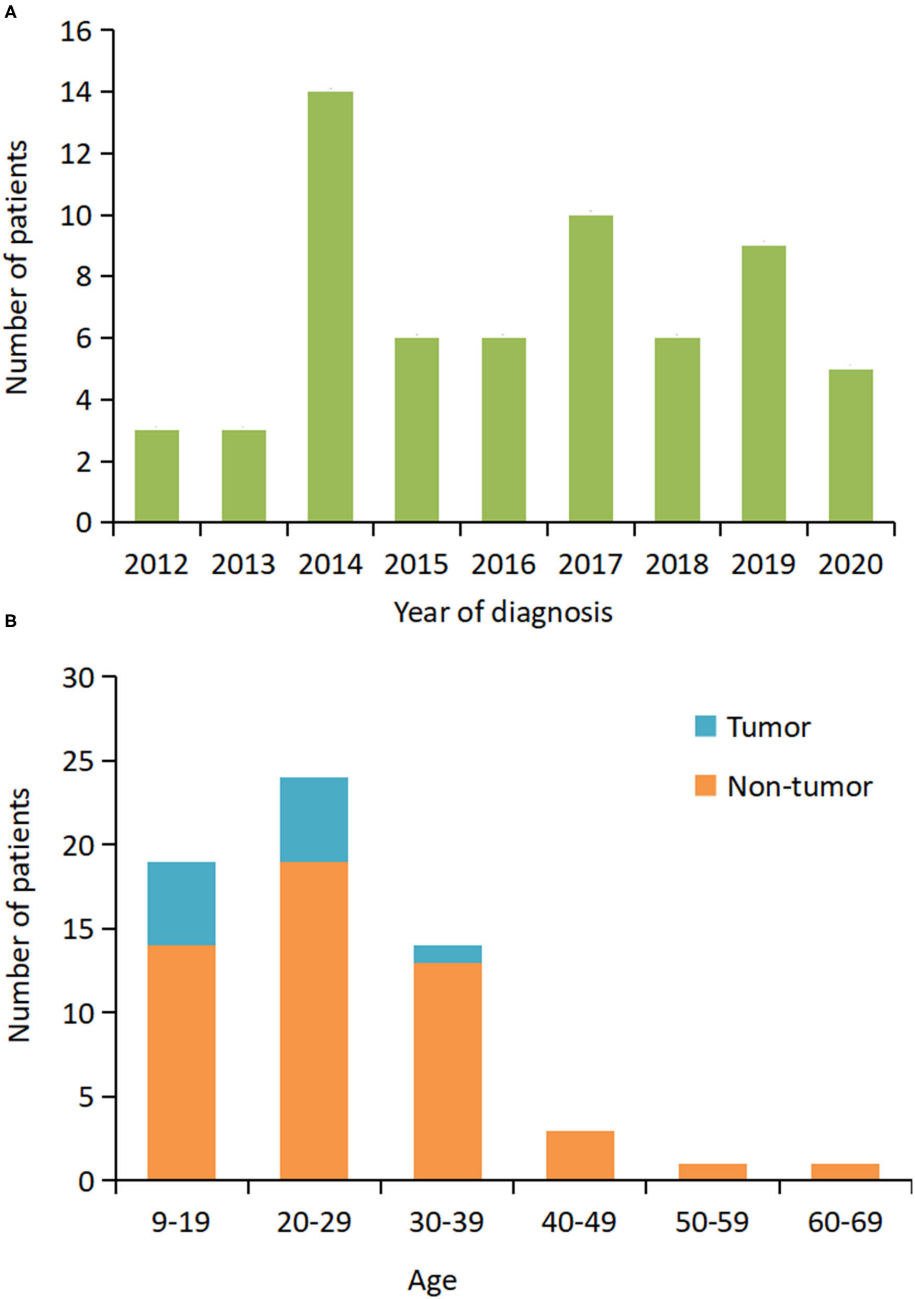
Distribution of patients who required mechanical ventilation based on the year of diagnosis and age. **(A)** The number of patients at the initial hospital visit from 2012 to 2020. **(B)** The number of patients by age and presence or absence of the tumor.

From June 2012 to September 2020, 305 patients with a definite diagnosis of anti-NMDAR encephalitis visited our hospital for treatment. Of these patients, we identified 62 (20.3%) patients that required MV (Table 1). Figure 1A shows that the number of patients increased over the study period from 2012 to 2020. The median age at disease onset was 26 years (interquartile range [IQR] 19–31 years); the peak number of patients requiring MV was between 20 and 29 years (Figure 1B). The median interpretation from anti-NMDAR encephalitis onset to MV was 15 (IQR 10–21) days. The main reasons for use of MV were decreased consciousness and/or SE (66%), acute respiratory failure (18%), and hypoventilation (5%). Intubation was performed in 43 (69%) patients and tracheotomy was performed in 28 (45%) patients.

**Table 1:** Baseline characteristics of the study cohort.

**Variable**	**Total patients**
	***n* = (62)**
Age, median (IQR), y	26 (19–31)
Male gender, (*n*/%)	32 (52)
Tumors, (*n*/%)	12 (19)
Delay between symptoms and MV, median (IQR), days	15 (10–21)
**Reason for MV, (** ***n*** **/%)**	
Decreased consciousness and/or status epilepticus	41 (66)
Respiratory failure	11 (18)
Hypoventilation	5 (8)
Others^a^	5 (8)
**Type of respiratory failure, (** ***n*** **/%)**	
Type I respiratory failure	41 (66)
Type II respiratory failure	21 (34)
**Cumulative symptoms, (** ***n*** **/%)**	
Coma	26 (42)
Status epilepticus	32 (52)
Refractory status epilepticus	11 (18)
Behavior dysfunction and/or cognitive deficits	60 (97)
Movement disorders	30 (48)
Speech disturbance	17 (27)
Autonomic dysfunction	39 (63)
Central hypoventilation	32 (52)
**Ancillary examination**	
CSF protein > 0.45 g/L, (*n*/%)	17 (27)
Pleocytosis, (*n*/%)	40 (65)
CSF NMDAR antibody titers ≥ 1:100, (*n*/%)	40 (65)
Abnormal MRI findings, (*n*/%)	26 (45)
Abnormal EEG findings, (*n*/%)	46 (78)
CRP, median (IQR), mg/L	12.7 (5.04–60.63)
IL-6, median (IQR), pg/ml	19.67 (8.54–38.18)
PCT, median (IQR), ng/ml	0.07 (0.05–0.26)
NLR, median (IQR)	5.34 (3.87–11.91)
Interval form onset to receive IT > 30 days, (*n*/%)	20 (32)
**First-line immunotherapy, (** ***n*** **/%)**	
IVIg only	11 (18)
MP only	6 (10)
IVIg + MP	45 (73)
PE	2 (3)
Second-line immunotherapy, (*n*/%)	5 (8)
**Complications, (** ***n*** **/%)**	
Severe pneumonia	11 (18)
Sepsis	5 (8)
Deep venous thrombosis	8 (13)
Gastrointestinal bleeding	19 (31)
Tracheostomy, (*n*/%)	28 (45)
Reintubation, (*n*/%)	5 (8)
Ventilator-associated pneumonia	4 (6)
ICU admission, (*n*/%)	23 (37)
Hospital length of stay, median (IQR), days	41 (23–63)
mRS score on admission, median (IQR)	5 (4–5)

*CSF, cerebrospinal fluid; CRP, C-reactive protein; EEG, electroencephalogram; ICU, admission intensive care unit; IL-6, interleukin-6; IQR, interquartile range; IT, immunotherapy; IVIg, intravenous immunoglobulins; MP, methylprednisolone; MRI, magnetic resonance imaging; mRS, modified Rankin Scale; MV, mechanical ventilation; NLR, neutrophil-to-lymphocyte ratio in peripheral blood; NMDAR, N-methyl-D-aspartate receptor; PE, plasma exchange; PCT, procalcitonin*.

a *Cardiac arrest (n = 1); severe hemodynamic instability (n = 2); aspiration (n = 2)*.

In the cohort during analysis, abnormal brain MRI, abnormal EEG, increased CSF protein, and pleocytosis were found in 26 of the 58 (45%), 46 of the 59 (78%), 17 of the 62 (27%), and 40 of the 62 (65%) patients, respectively. The tumor was detected in 12 (19%) patients. The tumor resection was performed in 11 out of 12 (92%) patients, and histology consisted of immature teratoma of the ovary (*n* = 2, 18%), mature teratoma of the ovary (*n* = 9, 82%). All patients received first-line immunotherapy, which included a combination of MP and IVIg (45/62, 73%), MP only (6/62, 10%), and IVIg only (11/62, 18%). Only five patients received second-line immunotherapy.

Additionally, two patients were excluded from analysis (non-invasive ventilation [NIV] vs. NIV + invasive ventilation [IV]; prolonged MV vs. short MV); one patient died within 15 days after MV; and in one patient, the duration of the ventilation was unknown due to the loss of follow-up. The median duration of MV was 15 days (IQR 7–32 days, range 1–386 days).

### Comparison Between the NIV and IV

**Figure 2:**
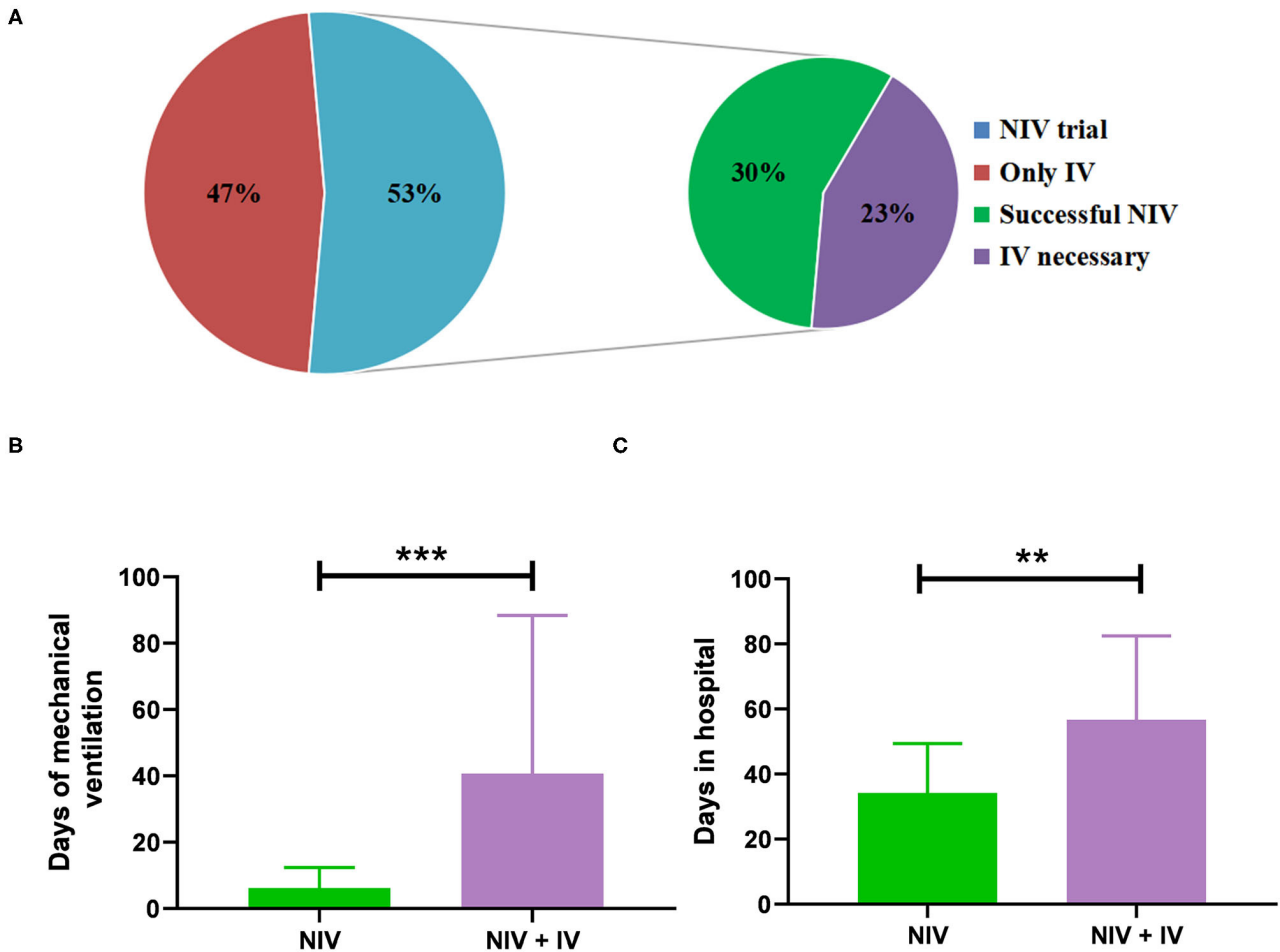
Administration of mechanical ventilation (MV). **(A)** The percentage of patients where non-invasive ventilation (NIV) was attempted before invasive ventilation (*n* = 32 out of 60) and the percentage of successful NIV trials and the percentage of NIV failure. Days of MV **(B)** and days of stay in the hospital **(C)** of patients treated with NIV alone (*n* = 18) as compared to patients treated with NIV and invasive ventilation (*n* = 14). Bars show mean ± SD. ***p < 0.001, **p < 0.01 (Mann-Whitney *U* test).

Non-invasive ventilation was performed in 32 patients and sufficed in 18 patients (30%) so that IV was not needed (Figure 2A). The duration of MV (median 5 days vs. 25 days; *p* < 0.01) and the length of stay in hospital (median 31 days vs. 51 days; *p* = 0.01) were significantly shorter in patients with NIV than in those who needed IV (Figures 2B,C). However, baseline characteristics did not differ significantly between the NIV and NIV + IV groups (Table 2). Furthermore, the NIV + IV did not significantly extend the duration of MV as compared to patients only with IV and without NIV trial (median 25 days vs. 20 days; *p* = 0.927).

**Table 2:** Comparison between the non-invasive ventilation and invasive ventilation.

**Variable**	**NIV (*n* = 18)**	**NIV + IV (*n* = 14)**	***P*-value**
Age, median (IQR), y	27 (18–30)	25 (17–29)	0.639
Male gender, (*n*/%)	8 (44)	7 (50)	1.000
Tumors, (*n*/%)	1 (6)	4 (29)	0.142
**Type of respiratory failure, (** ***n*** **/%)**			
Type I respiratory failure	11 (61)	12 (86)	0.235
Type II respiratory failure	7 (39)	2 (14)	
Delay between symptoms and MV, median (IQR), days	18 (12–26)	13 (10–17)	0.283
**Reason for MV, (** ***n*** **/%)**			
Decreased consciousness and/or status epilepticus	12 (67)	10 (71)	0.245
Respiratory failure	3 (17)	0 (0)	
Hypoventilation	3 (17)	2 (14)	
Others	0 (0)	2 (14)	
**Cumulative symptoms, (** ***n*** **/%)**			
Coma	4 (22)	7 (50)	0.142
Status epilepticus	10 (56)	7 (50)	1.000
Behavior dysfunction and/or cognitive deficits	18 (100)	13 (93)	0.437
Movement disorders	12 (67)	6 (43)	0.283
Speech disturbance	7 (39)	2 (14)	0.235
Autonomic dysfunction	11 (61)	9 (64)	1.000
**Ancillary examination**			
CSF protein > 0.45 g/L, (*n*/%)	4 (22)	5 (36)	0.453
Pleocytosis, (*n*/%)	10 (56)	11 (79)	0.266
CSF NMDAR antibody titers ≥ 1:100, (*n*/%)	11 (61)	9 (64)	1.000
Abnormal MRI findings, (*n*/%)	7 (44)	5 (36)	0.722
Abnormal EEG findings, (*n*/%)	12 (80)	11 (79)	1.000
CRP, median (IQR), mg/L	7.72 (1.54–60.4)	8.7 (4.96–54.45)	0.792
IL-6, median (IQR), pg/ml	23.12 (7.22–44.5)	20.29 (5.32–29.44)	0.884
PCT, median (IQR), ng/ml	0.16 (0.06–0.64)	0.06 (0.05–0.16)	0.207
NLR, median (IQR)	3.91 (2.68–6.68)	5.91 (4.49–11.67)	0.125
Interval form onset to receive IT > 30 days	8 (44)	4 (29)	0.471
**First-line immunotherapy, (** ***n*** **/%)**			
IVIg only	3 (17)	0 (0)	0.330
MP only	3 (17)	4 (29)	
IVIg + MP	12 (67)	10 (71)	
Second-line immunotherapy	2 (11)	2 (14)	1.000
**Complications, (** ***n*** **/%)**			
Severe pneumonia	1 (6)	1 (7)	1.000
Sepsis	1 (6)	0 (0)	NA
Deep venous thrombosis	0 (0)	3 (21)	NA
Gastrointestinal bleeding	5 (28)	5 (36)	0.712

*CSF, cerebrospinal fluid; CRP, C-reactive protein; EEG, electroencephalogram; IL-6, interleukin-6; IQR, interquartile range; IT, immunotherapy; IV, invasive ventilation; IVIg, intravenous immunoglobulins; MP, methylprednisolone; MRI, magnetic resonance imaging; MV, mechanical ventilation; NLR, neutrophil-to-lymphocyte ratio in peripheral blood; NMDAR, N-methyl-D-aspartate receptor; NIV, non-invasive ventilation; PCT, procalcitonin*.

### Comparison Between the Prolonged and Short MV

Twenty-seven (45%) of the ventilated patients needed prolonged MV. As shown in Table 3, patients with prolonged MV are significantly younger (*p* = 0.039), more often diagnosed with coma (*p* = 0.048), more often suffered from severe pneumonia (*p* = 0.008), have a higher proportion of tumor (*p* = 0.026), and a relatively longer length of the stay in the hospital (*p* < 0.001). Although no significance was found between the two groups, patients in the prolonged MV group had a substantially higher rate of RSE (*p* = 0.051) and central hypoventilation (*p* = 0.069) during the course of the disease as compared to short patients with MV. In addition, the need for prolonged MV was not associated with the type of respiratory failure, the reason for MV, abnormal MRI findings, and the CSF NMDAR antibody titers. Finally, although sepsis, deep venous thrombosis, and gastrointestinal bleeding were commonly observed in patients with MV > 15 days, these complications did not differ significantly between the two different groups.

**Table 3:** Characteristics in prolonged MV patients compared with short MV patients.

**Variable**	**MV ≤15 days (*n* = 33)**	**MV > 15 days (*n* = 27)**	***P*-value**
Age, median (IQR), y	28 (21–33)	20 (18–28)	**0.039**
Male gender, (*n*/%)	19 (58)	12 (44)	0.311
Tumors, (*n*/%)	3 (9)^a^	9 (33)^b^	**0.026**
Delay between symptoms and MV, median (IQR), days	16 (11–23)	13 (8–21)	0.109
Reason for MV, (n/%)			0.679
Decreased consciousness and/or status epilepticus	20 (61)	20 (74)	
Respiratory failure	6 (18)	4 (15)	
Hypoventilation	3 (19)	2 (7)	
Others^c^	4 (12)	1 (4)	
Type of respiratory failure, (*n*/%)			1.000
Type I respiratory failure	21 (64)	18 (67)	
Type II respiratory failure	12 (36)	9 (33)	
Cumulative symptoms, (*n*/%)			
Coma	10 (30)	15 (57)	**0.048**
Status epilepticus	14 (42)	17 (63)	0.113
Refractory status epilepticus	3 (9)	8 (30)	0.051
Behavior dysfunction and/or cognitive deficits	32 (97)	26 (93)	1.000
Movement disorders	15 (46)	14 (52)	0.622
Speech disturbance	11 (33)	6 (22)	0.342
Autonomic dysfunction	20 (61)	17 (63)	0.852
Central hypoventilation	13 (39)	17 (63)	0.069
**Ancillary examination**			
CSF protein > 0.45 g/L, (*n*/%)	11 (33)	5 (19)	0.255
Pleocytosis, (*n*/%)	21 (64)	18 (67)	0.807
CSF NMDAR antibody titers ≥ 1:100, (*n*/%)	21 (64)	18 (67)	0.807
Abnormal MRI findings, (*n*/%)	16 (52)	9 (36)	0.243
Abnormal EEG findings, (*n*/%)	25 (81)	19 (73)	0.498
CRP, median (IQR), mg/L	17 (4.8–64.5)	10.8 (5.1–52.3)	0.962
IL-6, median (IQR), pg/ml	20.1 (10.9–27.5)	17.2 (7.3–39.3)	0.867
PCT, median (IQR), ng/ml	0.08 (0.05–0.26)	0.07 (0.04–0.16)	0.535
NLR, median (IQR)	5.04 (3.11–11.35)	6.30 (4.74–14.31)	0.189
Interval form onset to receive IT > 30 days	12 (36)	8 (30)	0.582
**First-line immunotherapy, (** ***n*** **/%)**			
IVIg only	5 (15)	6 (22)	0.704
MP only	4 (12)	2 (7)	
IVIg + MP	24 (73)	19 (70)	
Second-line immunotherapy, (*n*/%)	2 (6)	3 (11)	0.649
**Complications, (** ***n*** **/%)**			
Severe pneumonia	1 (3)	8 (30)	**0.008**
Sepsis	1 (3)	3 (11)	0.318
Deep venous thrombosis	2 (6)	6 (22)	0.124
Gastrointestinal bleeding	7 (21)	11 (41)	0.156
Hospital length of stay, median (IQR), days	29 (21–44)	63 (39–80)	**<** **0.001**

*CSF, cerebrospinal fluid; CRP, C-reactive protein; EEG, electroencephalogram; IL-6, interleukin-6; IQR, interquartile range; IT, immunotherapy; IVIg, intravenous immunoglobulins; MP, methylprednisolone; MRI, magnetic resonance imaging; MV, mechanical ventilation; NLR, neutrophil-to-lymphocyte ratio in peripheral blood; NMDAR, N-methyl-D-aspartate receptor; PCT, procalcitonin*.

a *Mature teratomas of the ovary (n = 3)*.

b *Immature teratomas of the ovary (n = 2), mature teratomas of the ovary (n = 6)*.

c *Cardiac arrest (n = 1); severe hemodynamic instability (n = 2); aspiration (n = 2)*. *The bold values represent the p value < 0.05*.

### Follow-Up and Outcomes

The functional outcomes were assessable in 60 patients (median follow-up duration was 28 months, range 3–87 months). Overall, 77% (46/60) of the patients displayed a good neurological outcome (mRS ≤ 2) and long-term mortality was 13% (7/60). The proportion of patients with good neurological outcome was improved over the follow-up period (Supplementary Figure S1). Table 4 summarizes the predictors for poor neurological outcome in anti-NMDAR encephalitis patients who required MV at the 6-month follow-up. In univariate analysis, the prolonged MV (*p* = 0.02), the higher levels of CRP (*p* = 0.01), and NLR (*p* = 0.004) were closely associated with a poor neurological outcome at 6 months. Although there were no significant differences, the frequency of the tumor, coma, and SE was found to be higher in the poor neurological outcome subgroup. As complications during the acute phase might affect the prognosis of the disease, the incidence of complications (such as sepsis, severe pneumonia, deep venous thrombosis, and gastrointestinal bleeding) was compared, and it was observed found that all these complications occurred comparably between the two different groups (all *p* > 0.05).

**Table 4:** Predictors for the poor neurologic outcome (mRS > 2) at the 6-month follow-up.

**Potential predictors**	**mRS ≤2 at 6 months (*n* = 34)**	**mRS > 2 at 6 months (*n* = 20)**	***P*-value**
Age, median (IQR), y	26.5 (18.75–35)	24 (19–28)	0.23
Male gender, (*n*/%)	17 (50)	11 (55)	0.72
Tumors, (*n*/%)	5 (15)	6 (30)	0.29
Type of respiratory failure, (*n*/%)			0.25
Type I respiratory failure	24 (71)	11 (55)	-
Type II respiratory failure	10 (29)	9 (45)	-
Reason for MV, (*n*/%)			0.19
Decreased consciousness and/or status epilepticus	22 (65)	13 (65)	-
Respiratory failure	4 (12)	6 (30)	-
Hypoventilation	4 (12)	0 (0)	-
Others^a^	4 (12)	1 (5)	-
**Cumulative symptoms, (** ***n*** **/%)**			
Coma	12 (35)	10 (50)	0.22
Status epilepticus	16 (47)	10 (50)	0.84
Refractory status epilepticus	7 (21)	3 (15)	0.73
Behavior dysfunction and/or cognitive deficits	32 (94)	20 (100)	0.53
Movement disorders	15 (44)	10 (50)	0.68
Speech disturbance	9 (27)	7 (35)	0.51
Autonomic dysfunction	21 (62)	11 (55)	0.63
Central hypoventilation	19 (56)	7 (35)	0.14
**Ancillary examination**			
CSF protein > 0.45 g/L, (*n*/%)	11 (32)	4 (20)	0.37
Pleocytosis, (*n*/%)	24 (71)	12 (60)	0.43
CSF NMDAR antibody titers ≥ 1:100, (*n*/%)	22 (65)	13 (65)	1.0
Abnormal MRI findings, (*n*/%)	16 (49)	7 (41)	0.62
Abnormal EEG findings, (*n*/%)	25 (78)	13 (68)	0.44
CRP, median (IQR), mg/L	8.52 (4.68–46.58)	38.4 (14.3–79.3)	**0.01**
IL-6, median (IQR), pg/ml	20.1 (7.94–37.91)	29.8 (14.05–70.11)	0.27
PCT, median (IQR), ng/ml	0.08 (0.05–0.19)	0.07 (0.05–0.43)	0.96
NLR, median (IQR)	4.88 (3.06–7.61)	9.6 (5.12–17.25)	**0.004**
Interval form onset to receive IT > 30 days	13 (38)	5 (25)	0.38
**Complications, (** ***n*** **/%)**			
Severe pneumonia	3 (9)	6 (30)	0.06
Sepsis	1 (3)	2 (10)	0.55
Deep venous thrombosis	3 (9)	2 (10)	1.00
Gastrointestinal bleeding	6 (18)	8 (40)	0.07
Prolonged MV, (*n*/%)	11 (32)	13 (65)	**0.02**

*CSF, cerebrospinal fluid; CRP, C-reactive protein; EEG, electroencephalogram; IL-6, interleukin-6; IQR, interquartile range; IT, immunotherapy; IVIg, intravenous immunoglobulins; MP, methylprednisolone; MRI, magnetic resonance imaging; MV, mechanical ventilation; NLR, neutrophil-to-lymphocyte ratio in peripheral blood; NMDAR, N-methyl-D-aspartate receptor; PCT, procalcitonin*.

a *Cardiac arrest (n = 1); severe hemodynamic instability (n = 2); aspiration (n = 2)*. *The bold values represent the p value < 0.05*.

**Figure 3:**
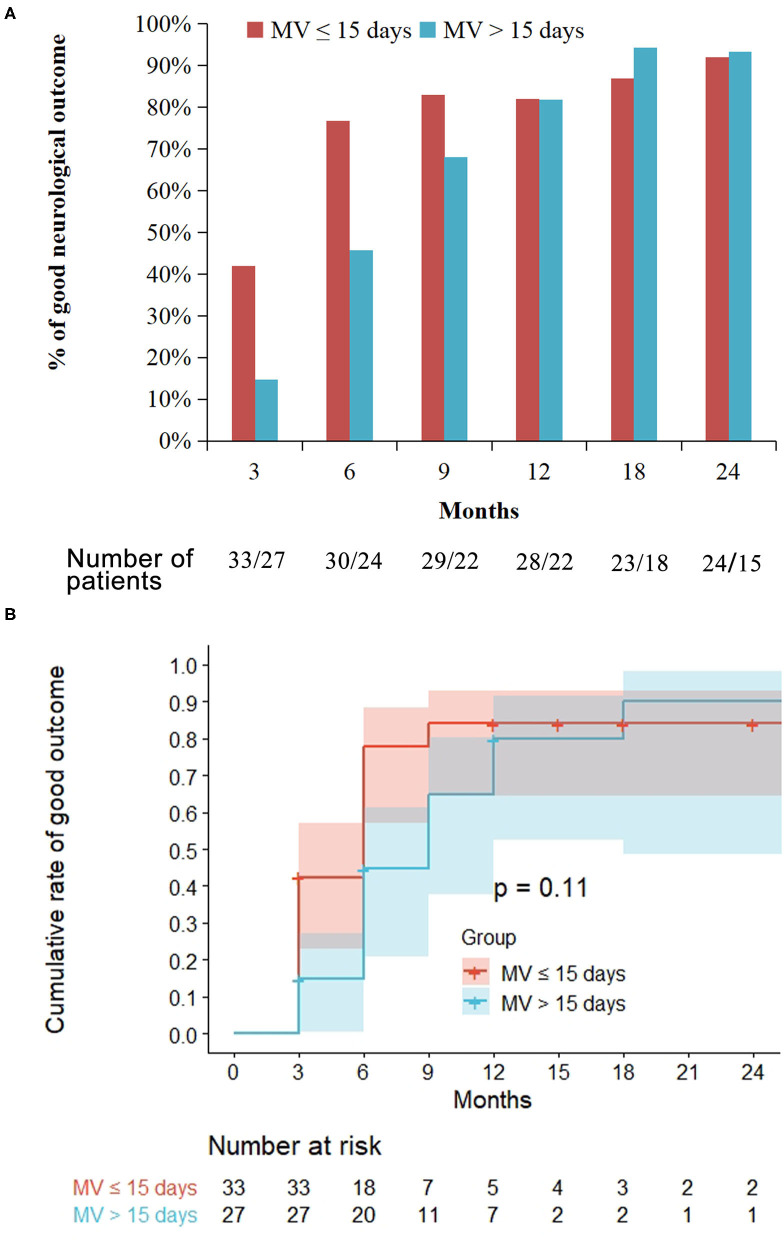
Treatment outcome in the prolonged mechanical ventilation (MV) group as compared with the short MV group. Prolonged MV is defined as MV >15 days while short MV is defined as MV ≤ 15 days. Good outcome is defined as a modified Rankin Scale score ≤ 2. **(A)** At the 3 and 6 months' follow-up, the proportion of patients that achieved a good outcome in the prolonged MV group were significantly lower than that in the short MV group (*p* = 0.025 and *p* = 0.02, respectively). **(B)** Kaplan-Meier curve showed similar probability of long-term good outcomes among the prolonged and the short MV patients.

Of the 33 patients with short MV, the proportion of patients with good neurological outcomes was 42 (14/33), 77 (23/30), 82 (23/28), and 92% (22/24) at 3, 6, 12, and 24 months' assessment, respectively. However, in the 27 patients with prolonged MV, the proportion of patients with good neurological outcomes was 15 (4/27), 46 (11/24), 82 (18/22), and 93% (14/15) at 3, 6, 12, and 24 months' assessment, respectively. At the 3 and 6 months assessment, the proportion of patients that exhibited good outcomes in the prolonged MV group was significantly lower than that in the short MV group (*p* = 0.025 and *p* = 0.02, respectively, Figure 3A). Moreover, there were no significant differences noted in the neurological outcomes between the prolonged MV and short MV groups after 6 months' treatment. Kaplan-Meier curve analysis clearly demonstrated a higher trend of good neurological outcome in patients with short MV, but there was no statistical significance observed between the two groups (Figure 3B).

The comparison of clinical outcomes of the ventilated patients has been shown in (Supplementary Table S1). During the follow-up period, patients with prolonged MV needed a substantially longer time to achieve good neurological outcome than patients with short MV (median duration 6 months vs. 3 months, *p* = 0.004), which is due to residual deficits of anti-NMDAR encephalitis. Despite the delayed recovery in the prolonged MV group being found based on the mRS evaluation in this study, a comprehensive neuropsychological evaluation was not performed. Moreover, there were no significant differences noted between patients with short MV and patients with prolonged MV regarding mortality, relapse rate, and the follow-up time. Finally, in patients with prolonged MV, four patients received repeated cranial MRI examinations (median, 12.5 months; range, 4–31 months), and all of these patients were without any changing area of brain lesions. In patients with short MV, 13 patients received repeated cranial MRI examinations (median, 9 months; range, 1–23 months): 11 of these without any changing area of brain lesions, one repeat MRI brain scan showed gradually evolving brain atrophy, and one patient's lesions resolved after treatment (Supplementary Figure S2). There was also no significant difference between both groups regarding follow-up imaging.

## Discussion

The need for MV is an indicator for a more severe course but MV is no treatment of anti-NMDAR encephalitis but just treatment of symptoms (coma, respiratory failure, and hypoventilation) that are caused by anti-NMDAR encephalitis. For the first time, we have described patients with anti-NMDAR encephalitis who requiring the MV. We found that (1) the most common reasons for MV were decreased consciousness and/or SE; (2) upon comparison with patients with short MV, patients with prolonged MV were significantly younger, displayed a higher frequency of coma, tumors, and severe pneumonia, and with a longer length of stay in the hospital; (3) during the follow-up, most anti-NMDAR encephalitis patients who required MV eventually achieved good neurological outcome; (4) although patients in the prolonged MV group took longer time to achieve the good neurological outcome, there was no statistical difference in the long-term prognosis, mortality, and recurrence between the prolonged and the short MV groups.

To date, there is only limited information about the application of MV in patients with anti-NMDAR encephalitis beyond aggressive immunosuppression. Overall characteristics of our patient cohort did not differ significantly from the previous reports related to anti-NMDAR encephalitis with regard to the sex, age, abnormal brain MRI, abnormal EEG, increased CSF protein pleocytosis, the prevalence of the tumors, and the long-term mortality (2, 3, 22). However, the frequency of coma (42%) and SE (52%) in our study was significantly higher than in the cohorts of unselected patients with anti-NMDAR encephalitis (16% coma and 30% SE) (22), which may be attributed to the fact that the presence of these clinical signs could possibly be the predictors for the administration of MV therapy. However, due to the limited availability of the medical recourses and high hospitalization costs, the ICU admission rate was underestimated in this study. This phenomenon has been described in detail in the previous article on Chinese anti-NMDAR encephalitis patients (3, 22).

Similar to the previous reports about the AE, the most common reasons for MV in our study were decreased consciousness and/or SE (4), which was similar to the results obtained in other neurological diseases, such as acute ischemic stroke, intracranial hemorrhage, and subarachnoid hemorrhage (23). Previous studies have hypothesized that the reason for MV could possibly serve as a predictor in acute stroke patients (23). However, in this study, the reason for MV was not associated with the functional outcome. The median duration of MV was 15 days in this study, which was significantly shorter than the duration of MV in the patients between 2008 and 2014 (median 47 days) (20). The difference may be due to the following two major facts: firstly, we enrolled NIV patients, and these patients are often treated with shorter duration of MV (13); secondly, awareness for the disease and treatment strategies have markedly changed over the time which might also significantly influence the results (24).

Our study first described the different clinical characteristics of NIV in patients with anti-NMDAR encephalitis. Importantly, we did not observe a longer duration of MV when NIV was not successful. By contrast, it was found that a relatively shorter duration of MV and the length of stay in the hospital when NIV were successful. Thus, we hypothesized that NIV could be considered in appropriate cases before giving IV. However, owing to the small number of events analyzed, the various factors for NIV failure could not be determined. Further investigations in this area are hence required in the future.

Notably, there was a trend toward a higher frequency of the tumor and coma in the prolonged MV group. Previous studies have also suggested that the presence of tumor and coma was associated with the clinical severity in anti-NMDAR encephalitis (20, 22, 25, 26). Therefore, these parameters might serve as important factors in patients with anti-NMDAR encephalitis who required prolonged MV. We also noticed that anti-NMDAR encephalitis patients in the prolonged MV group were significantly younger than the patients in the short MV group. We are unable to speculate the exact reasons why younger patients require a longer duration of MV, but this result may be related to the selection bias and the sample sizes. In our study, prolonged patients with MV exhibited a significantly higher incidence of combined severe pneumonia. Severe pneumonia is highly prevalent in critically ill patients and has been reported to be one of the most common causes of death in anti-NMDAR encephalitis. This finding indicated that severe pneumonia in anti-NMDA receptor encephalitis requires intensive supportive management. In addition, no significant difference was found in sepsis, deep venous thrombosis, and gastrointestinal bleeding between the two different groups, which have been previously reported to be associated with the prolonged MV (23, 27), however, because of the small size analyzed in this cohort, the results should be interpreted cautiously.

In addition, similar to patients with critically ill diseases other than anti-NMDAR encephalitis, prolonged MV has been associated with a longer length of stay in the hospital and a slower recovery process (13, 28). However, most survivors could regain the independent capacity to walk and live at the end of the treatment. A number of previous clinical studies have also indicated that even severely affected patients with different neurological diseases [such as myasthenia gravis (13) and Guillain–Barré syndrome (28)] who required prolonged MV may show better clinical recovery. Strikingly, in this cohort, the need for prolonged MV was associated with the short-term outcome but not found to be related to the mortality, relapse, and poor functional long-term outcomes. This phenomenon was found to be similar to published literature about critically ill and ICU-bound anti-NMDAR encephalitis (20). Thus, prolonged MV following the disease may be a possible predictor of the short-term but not that of long-term prognosis.

In this study, prolonged MV, higher levels of CRP and NLR were found to be closely associated with a poor functional outcome at 6 months. Previous studies have also reported similar results in acute encephalitis of various etiologies, such as AE (4). In addition, the incidence of severe pneumonia and gastrointestinal bleeding was significantly higher in the group that displayed poor functional outcomes at 6 months, although there were no significant differences. We speculate that the short-term outcomes of patients with anti-NMDAR encephalitis who required MV might be associated with the possible occurrence of complications, which is consistent with ICU-bound AE (4). Surprisingly, coma, RSE pleocytosis, CSF NMDAR antibody titers, and abnormal MRI findings were not associated with the poor functional outcome at 6 months, however, as a result of the specificity of the study population and the small size of our cohort, the prognostic significance of these various important factors requires detailed investigations.

This study has several major limitations that should be considered carefully. Firstly, the study is a potentially retrospective analysis, which may lead to significant bias. Secondly, based on the retrospective features, it was not feasible to detect various early predictors which could indicate the need for requiring MV, but it is a precursor to the various prospective and standardized studies related to this topic. Thirdly, because of the data unavailability, we could not accurately account for the pulmonary function tests before the ventilation and ventilator settings. Fourthly, anti-NMDAR encephalitis is relatively a rare disease and we should be extremely careful in interpreting our results due to the relatively small size of this study. Fifthly, in addition to the mRS score, other detailed evaluation parameters (such as cognitive or behavioral function, health-related quality of life, or post-traumatic stress disorder) are needed to completely evaluate the long-term outcomes of anti-NMDAR encephalitis requiring MV.

In summary, this study specifically focused on the analysis of anti-NMDAR encephalitis patients who required MV. We found that majority of anti-NMDAR encephalitis patients who required MV eventually achieved favorable long-term outcomes after appropriate immunotherapy, despite the longer duration of MV. Overall, our results may potentially help clinicians in routinely implementing the specialized ventilator management of severe anti-NMDAR encephalitis patients to achieve optimal treatment outcomes.

## Data Availability Statement

The raw data supporting the conclusions of this article will be made available by the authors, without undue reservation.

## Ethics Statement

The studies involving human participants were reviewed and approved by the Research Ethics Committee of West China Hospital of Sichuan University. The patients/participants provided their written informed consent to participate in this study.

## Author Contributions

JLin and QX: carried out the statistical analysis and drafted the manuscript. XL: collected and interpreted the data. JLi: conceptualized, designed the study, and revised the manuscript. All authors contributed to the article and approved the submitted version.

## Funding

This study was supported by the National Natural Science Foundation of China (grant 82071459).

## Conflict of Interest

The authors declare that the research was conducted in the absence of any commercial or financial relationships that could be construed as a potential conflict of interest.

## Publisher's Note

All claims expressed in this article are solely those of the authors and do not necessarily represent those of their affiliated organizations, or those of the publisher, the editors and the reviewers. Any product that may be evaluated in this article, or claim that may be made by its manufacturer, is not guaranteed or endorsed by the publisher.
