# Supplementary material for: Risk Factors and Prognosis in Patients With Anti-N-Methyl-D-Aspartate Receptor Encephalitis Requiring Prolonged Mechanical Ventilation
Source: Front Neurol. 2022 Feb 9;13:814673. doi: 10.3389/fneur.2022.814673 (PMC8863869; doi:10.3389/fneur.2022.814673)
Supplement: Supplementary file 1 [file Data_Sheet_1.pdf]

Risk factors and prognosis in patients with anti-N-methyl-D-aspartate receptor encephalitis requiring prolonged mechanical ventilation

I. eResults

Table 1 Functional outcome in prolonged MV patients compared with short MV patients

**Variable**	**MV ≤ 15 days (n = 33)**	**MV > 15 days (n = 27)**	**P value**
Follow-up time, median (IQR), months	25 (13-60)	32 (12-54)	0.688
Good outcome during follow-up, (n/%)	27（82）	19 (70)	0.297
Time to achieve good outcome, median (IQR), months	3 (3-6)	6 (6-9)	**0.004**
Mortality, (n/%)	2 (6)	5 (19)	0.226
Relapse, (n/%)	6 (18)	1 (4)	0.116
mRS score at last follow-up			
0	18 (55)	10 (37)	0.152
1	4 (12)	6 (22)	
2	5 (15)	3 (11)	
3	4 (12)	0 (0)	
4	0 (0)	2 (7)	
5	0 (0)	1 (4)	
6	2 (6)	5 (19)	

IQR: interquartile range; mRS: modified Rankin Scale; MV: mechanical ventilation.

II. eFigures

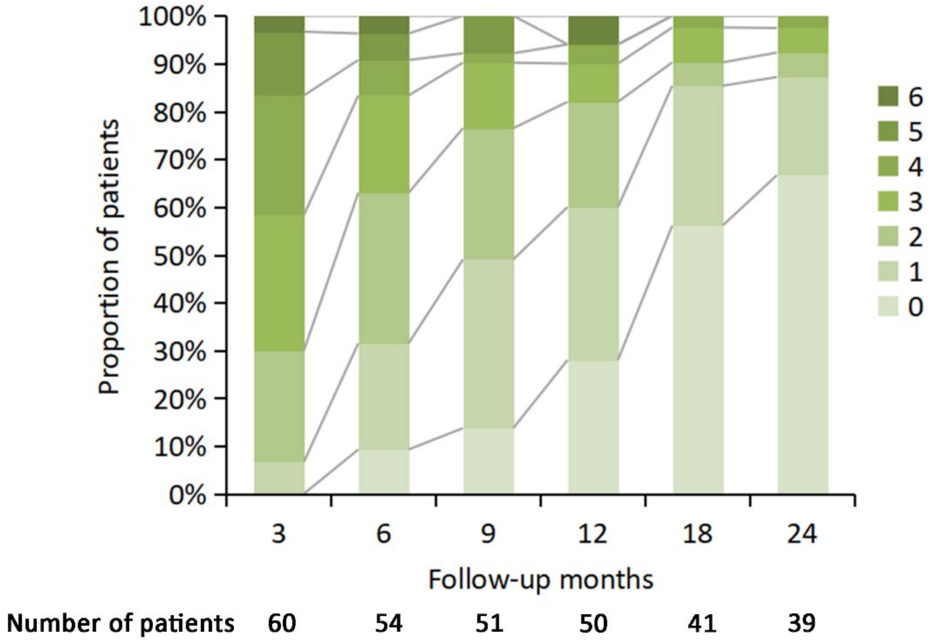

100%
90%
80%
70%
60%
50%
40%
30%
20%
10%
0%
Proportion of patients
3
6
9
12
18
24
Follow-up months
Number of patients
60
54
51
50
41
39
6
5
4
3
2
1
0

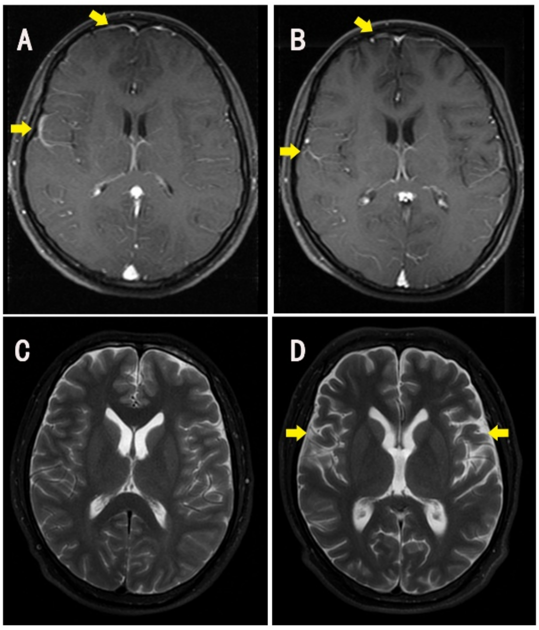

A
B
C
D
